# *In vitro* ruminal fermentation and cow-to-mouse fecal transplantations verify the inter-relationship of microbiome and metabolome biomarkers: potential to promote health in dairy cows

**DOI:** 10.3389/fvets.2023.1228086

**Published:** 2023-08-17

**Authors:** Jui-Chun Hsieh, Shih-Te Chuang, Yu-Ting Hsu, Shang-Tse Ho, Kuan-Yi Li, Shih-Hsuan Chou, Ming-Ju Chen

**Affiliations:** ^1^Department of Animal Science and Technology, National Taiwan University, Taipei City, Taiwan; ^2^Department of Veterinary Medicine, College of Veterinary Medicine, National Chung Hsing University, Taichung City, Taiwan; ^3^Department of Wood Based Materials and Design, National Chiayi University, Chiayi City, Taiwan; ^4^Graduate Institute of Biomedical and Pharmaceutical Science, Fu-Jen Catholic University, New Taipei City, Taiwan; ^5^Biotools Co. Ltd., New Taipei City, Taiwan

**Keywords:** *Ruminococcus flavefaciens*, *Bifidobacterium longum* subsp. longum, metabolites, biomarkers, dairy cows

## Abstract

**Introduction:**

There are differences in the gut microbiome and metabolome when the host undergoes different physical or pathological conditions. However, the inter-relationship of microbiome and metabolome biomarkers to potentially promote the health of dairy cows needs to be studied. Further, the development of next-generation probiotics for dairy cattle health promotion has not been demonstrated.

**Objective:**

In the present study, we identified the microbiome and metabolome biomarkers associated with healthy cows.

**Methods:**

We analyzed the relationships of the ruminal microorganism profile and metabolites between healthy and mastitis lactating dairy cows. The roles of bacterial biomarker were further verified by in vitro fermentation and cow-to-mouse fecal microbiota transplantation (FMT).

**Results:**

Two species, *Ruminococcus flavefaciens* and *Bifidobacterium longum* subsp. *longum*, and six rumen metabolites were positively correlated with healthy cows by Spearman’s correlation analysis. Through in vitro ruminal fermentation, inoculating *R. flavefaciens* and *B. longum* subsp. *longum* showed the upregulation of the levels of putrescine, xanthurenic acid, and pyridoxal in the mastitis ruminal fluid, which confirmed the inter-relationships between these microbiota and metabolites associated with healthy cows. Further, we verified the role of *R. flavefaciens* and *B. longum* subsp. *longum* in promoting health by FMT. The administration of *R. flavefaciens* and *B. longum* subsp. *longum* reduced the death rate and recovered the bodyweight loss of germ-free mice caused by FMT mastitis feces.

**Discussion:**

We provided evidence that the bacterial biomarkers alter downstream metabolites. This could indirectly indicate that the two bacterial biomarkers have the potential to be used as next-generation probiotics for dairy cattle, although it needs more evidence to support our hypothesis. Two species, *R. flavefaciens* and *B. longum* subsp. *longum*, with three metabolites, putrescine, xanthurenic acid, and pyridoxal, identified in the ruminal fluid, may point to a new health-promoting and disease-preventing approach for dairy cattle.

## 1. Introduction

Bovine rumen possesses a highly diverse population of microorganisms, including bacteria, protozoa, archaea, and fungi, which degrade and ferment the plant materials into digestible compounds (1). Proteobacteria, Firmicutes, and Bacteroidetes are the dominant phyla of the Kingdom Eubacteria in dairy cattle rumen (2, 3). However, the bacterial abundance in the digestive tract may fluctuate because of age, nutrients, and other factors related to lifestyle (4–6) or immune status (7). Changes in the gut microbiota play an influential role in the performance of the animals (3, 8) as well as contribute to the development of diseases including mastitis (7, 9). Gut dysbiosis also plays an important role in the health of dairy cows due to the difference and diversity of the gastrointestinal microbiota that competes for nutrients, regulate the immune system, and produce metabolites (10). Different microbiota found in feces (9) and ruminal fluid (7) of healthy and mastitis cows, and induction of mastitis in germ-free (GF) mice by fecal microbiota transplantation (FMT) suggested that bovine mastitis is not necessary a local infection of the mammary glands (9). Many evidences have shown that the stress factors such as high concentrate feeding or heat stress, could disturb the rumen microbiota and upregulate the level of lipopolysaccharide (LPS) (11), resulting in changes in the permeability of the rumen epithelial layer (12). The rumen-derived LPS could enter the mammary gland via blood circulation and further impair the blood-milk barrier, leading to inflammation of the mammary gland in cows (12, 13). Additionally, Zhao et al. (13) suggested that ruminal dysbiosis-derived low-grade endotoxemia could cause mastitis and worsen pathogen-induced mastitis by damaging host anti-inflammatory enzymes.

The metabolites, derived from fermentation by rumen microorganisms, are considered as a downstream outcome, illustrating the interaction between microorganisms, hosts, and the microenvironment. Metabolomics has been employed to evaluate the quality of milk (14) and search for new biomarkers for disorders (7). Chuang et al. (7) identified seven rumen fluid metabolites that changed between healthy and mastitis cows, which could be used as potential biomarkers for the diagnosis of mastitis.

The probiotics currently available to farm animals are generally limited to a narrow range of organisms. Characterizing the gut microbiota and metabolites is a novel preventive or therapeutic approach for the development of next-generation probiotics (15, 16). Therefore, the comprehensive description of the ruminal microbiota and metabolome and their roles in health and disease is crucial. Although the ruminal microbial and metabolomic structure in lactating dairy cows with mastitis has been studied (7), the inter-relationship of the microbiome and metabolome biomarkers in promoting health in dairy cows has never been confirmed. The role of bacterial biomarkers in health promotion and disease prevention also remains unknown. Thus, this study first identified the microbiome and metabolome biomarkers associated with healthy cows by evaluating the relationships among the ruminal microbial profile, metabolites, and mastitis outcomes. We then verified the inter-relationship of the microbiome and metabolome biomarkers and the role of bacterial biomarkers in health promotion and disease prevention through *in vitro* ruminal fermentation and cow-to-mouse fecal microbiota transplantation, respectively.

## 2. Materials and methods

### 2.1. Potential ruminal microorganism biomarkers and related metabolites for healthy dairy cows

#### 2.1.1. Animals and sample collection

Thirty lactating Holstein dairy cows with 120–240 milk production days and an average age of 3.53 ± 0.67 years from a commercial farm were involved in the present study. All cows were under the same management, receiving total mixed ration (TMR) feeding and water *ad libitum*, and milked twice per day. After the outcomes of veterinary diagnosis, raw milk test with California mastitis test (CMT) and somatic cell counts (SCC), and serum proinflammatory cytokines, 15 healthy cows and 15 cows with clinical mastitis were selected for microbiomic and metabolomic analysis. Cows with one quarter milk showed positive reaction by CMT, SCC ≥ 1,000,000 cells/mL, and elevated cytokines in serum were defined as mastitis cows. On the other hand, cows with negative CMT reaction, SCC < 200,000 cells/mL, and no specific cytokines were included in health group (17). Milk, ruminal fluid, and blood samples were collected 2 h after morning feeding (4 h after morning milking), according to previously described methods (7).

#### 2.1.2. Analysis of somatic cell counts and N-acetyl-β-D-glucosaminidase in milk

The California mastitis test kit (ImmuCell Corp., Portland, ME, United States) was used to analyze milk CMT reaction on the farm and was followed the manufacturer’s instructions. The SCC of quarter milk samples was conducted by a Fossomatic FC instrument (Foss Electric, Hillerød, Denmark). Milk N-acetyl-β-D-glucosaminidase (NAGase) activity was measured with a fluoro-optical method described by Kalmus et al. (18).

#### 2.1.3. Analysis of serum cytokines

The commercial enzyme-linked immunosorbent assay kits (Bovine TNF-alpha and IL-6 DuoSet ELISA kit, R&D Systems, Minneapolis, MN, United States) were used to measure the levels of tumour necrosis factor (TNF)-α and interleukin (IL)-6.

#### 2.1.4. Microbiome analysis

Total genomic DNA was extracted from ruminal fluid samples using the bead-beating method (19). The microbiome analysis adopted the method described by Chuang et al. (7) using the Illumina HiSeq 2,500 PE250 platform (20–25). The representative sequence for each operational taxonomic units (OTUs) was analyzed through taxonomic annotation (26, 27) and determined the alpha diversity (Chao1 richness estimator and Shannon’s diversity index). We used partial least squares discriminant analysis (PLS-DA), and the linear discriminant analysis (LDA) effect size (LEfSe) algorithm to analyze the data (28). The false discovery rate (FDR) was used to carry out multiple testing for the correction of the *p* value using the Benjamini–Hochberg procedure.

#### 2.1.5. Metabolite analysis

The ruminal fluid sample preparation and metabolite analysis adopted the method described by Chuang et al. (7). The orthogonal PLS-DA (oPLS-DA) model with MetaboAnalyst 5.0^1^ (29) and the Kyoto Encyclopedia of Genes and Genomes (KEGG) pathways using the KEGG database were also analyzed.

### 2.2. Verification of potential biomarkers related to the health of dairy cows

#### 2.2.1. Bacterial preparation

*Bifidobacterium longum* subsp. *longum* (BCRC 14664) and *Ruminococcus flavefaciens* (DSM 25089) were purchased from the Bioresource Collection and Research Center (BCRC, Food Industry Research and Development Institute, Hsinchu, Taiwan) and Deutsche Samulung von Mikroorganisem und Zelkultruen (DSMZ, Braunscheig, Germany), respectively. *B. longum* subsp. *longum* was activated three times using the Lactobacilli MRS broth (Lactobacilli de Man, Rogosa and Sharpe broth, Acumedia, Lansing, MI, United States) at 37°C before subsequent analysis. *R. flavefaciens* was cultured under an anaerobic environment at 37°C using the medium formulated by Wang (30).

#### 2.2.2. *In vitro* ruminal fermentation

After the outcomes of veterinary diagnosis, CMT, SCC, NAGase, and serum proinflammatory cytokines, 5 healthy cows (H group) and 5 cows with clinical mastitis (M group) under a similar milk production stage (the first 40- to 60 day lactation) and an average age of 2.75 ± 0.71 years old from same commercial farm mention above were selected as rumen fluid donors. After the morning feeding, 1,500 mL of rumen fluid was obtained as described above. The samples were filtered with four layers of cheesecloth and placed in a flask with thermal insulation (39°C) before being transported to the laboratory. The artificial saliva, prepared according to the description of Menke and Steingass (31), was combined with rumen fluid in a non-oxygen atmosphere. The 40 mL mixture was filled into a 100 mL serum bottle with CO_2_ containing 0.4 mg of feed subtract (fresh TMR prepared by the farm) and bacterial culture. Both the H and M groups of rumen fluid were further divided into 4 sub-groups defined as followed: A, with 1 mL of sterile ddH_2_O as the control; B, with 1 mL of *B. longum* subsp. *longum* (10^6^ CFU/mL) bacterial culture; C, with 1 mL of *R. flavefaciens* (10^6^ CFU/mL) bacterial culture; D, with 1 mL of each bacterial culture. For fermentation, the bottles were capped and incubated in a shaking incubator (120 rpm) at 39°C. The fermented fluid was collected at 0, 3, and 12 h.

#### 2.2.3. Qualitative metabolites

The ruminal fluid samples were centrifuged at 13,400 × g for 15 min. The supernatants were analyzed using a Shimadzu LC-20A high-performance liquid chromatography (HPLC) system (Shimadzu, Kyoto, Japan) coupled to a linear ion trap-Orbitrap mass spectrometer (LTQ Orbitrap Velos, Thermo Fisher Scientific, Waltham, MA, United States). The standards of metabolites were used for the qualitative analysis.

#### 2.2.4. Fecal microbiota transplantation

Fresh fecal samples from 15 mastitis and 15 healthy cows which were the same as Section 2.1.1. mentioned were, respectively, collected. The preparation procedure followed the method described by Ma et al. (9). GF mice were obtained and housed according to animal care regulations in the germ-free animal facility at the Animal Resource Center, National Taiwan University (Taipei, Taiwan). A total of 15 female adult (8 week-old) C57BL/6 J mice were randomly divided into three groups, which received 0.3 mL fecal supernatant from (i) healthy cows (Control group), (ii) mastitis cows (Mastitis group), or (iii) mastitis cows, plus 10^8^ CFU per day of *B. longum* subsp. *longum* and *R. flavefaciens* administration (M + BR group) for 4 weeks. The animals had measured bodyweight per week to determine the changes in body weight during the experiment period. The three groups of mice were caged in different gnotobiotic isolators after FMT to prevent cross-contamination.

### 2.3. Statistical analysis

All phenotypic and next-generation sequencing (NGS) data were analyzed with a nonparametric Mann–Whitney U test to identify significant differences between groups. Spearman’s correlation analysis was used to conduct the correlation between the relative abundance of biomarkers and metabolites. Statistical Analysis System v9.4 (SAS Institute Inc., Cary, NC, United States) and R software were used for all statistical analysis.

## 3. Results

### 3.1. Healthy status of tested dairy cows

**Figure 1:**
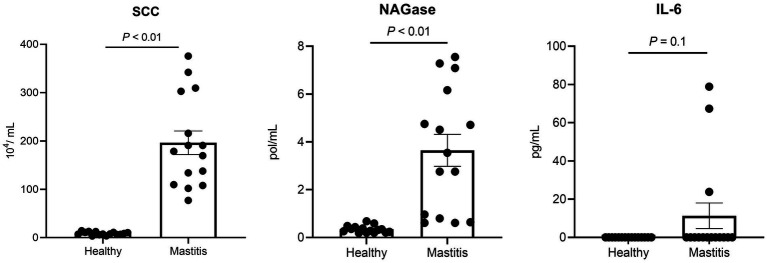
Milk somatic cell counts (SCC), NAGase, and serum IL-6 of healthy and mastitis cows.

Since the healthy status is crucial for this study, the mastitis cows were selected not only by veterinary diagnosis and CMT, but also by the milk SCC and NAGase as well as serum proinflammatory cytokines. The selected 15 mastitis cows demonstrated significantly higher milk SCC (*p* < 0.05) and NAGase (*p* < 0.05) than those of the 15 healthy counterparts (Figure 1). The serum IL-6 in the mastitis cows was also higher than that of healthy cows, which provided a solid foundation for the current study.

### 3.2. Beta diversity illustrates the dissimilar gut microbiota harbored in ruminants between healthy and mastitis cows

**Figure 2:**
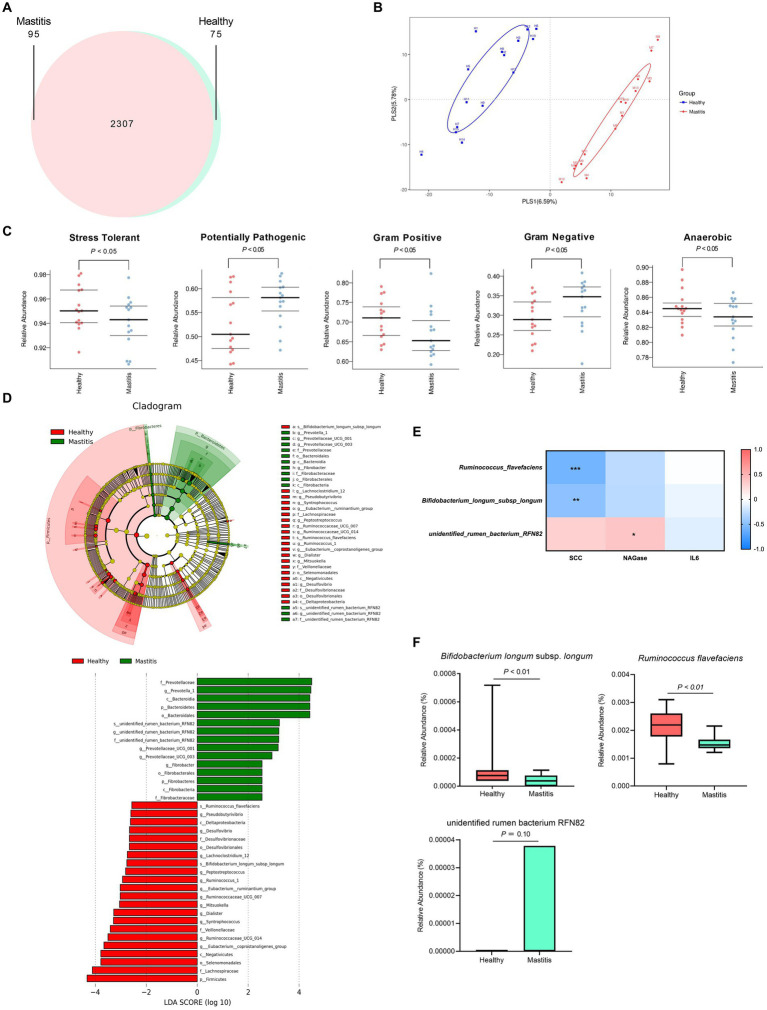
Ruminal bacteria and archaea composition identified by 16S rRNA sequencing of healthy and mastitis cows. **(A)** Venn diagram illustrating 2,307 operational taxonomic units (OTUs) of core microbiota identified both in healthy and mastitis cows. **(B)** Partial least squares discriminant analysis (PLS-DA) plot based on the relative abundance of OTUs indicates a significantly different composition of healthy versus mastitis cows. Ellipses represent 95% confidence intervals for each group. **(C)** Predicted phenotypes. **(D)** Significant differential biomarkers were identified using the LEfSe algorithm. **(E)** Spearman’s correlation test between mastitis markers and gut microbial biomarkers at the species level. Each cell was colored corresponding to the Spearman’s correlation results. Significant difference: *p < 0.05 and **p < 0.01. **(F)** Significant relative abundance of differential biomarkers.

After verifying the healthy status of the cows, we analyzed the ruminal microbiota by NGS. 16S rRNA analysis revealed a total of 778,270 and 794,717 effective tags from 1,200,157 and 1,233,089 raw paired-end reads from healthy and mastitis groups, respectively. The Venn diagram in Figure 2A showed that 2,307 OTUs were identical between the groups, with 75 and 95 unique OTUs for the healthy and mastitis groups, respectively. The alpha diversity (Chao1 richness estimator and Shannon’s diversity index) revealed no significant difference (*p* > 0.05) between the two groups (Supplementary Figure S1). The top 10 dominant taxa at the genus level, which covered 58% of the total genus level results, were identical between groups but with different proportions (Supplementary Figure S2). Additional beta diversity analysis separated the healthy and mastitis groups using the PLS-DA plot (Figure 2B). PLS1 and PLS2 explained 6.59 and 5.78%, respectively, of the variation in gut microbiota composition, illustrating the dissimilar gut microbiota harbored in ruminants. The predicted phenotypes showed that the healthy group possessed higher relative abundance in stress-tolerant, anaerobic, and Gram-positive bacteria, and lower abundance in Gram-negative and potentially pathogenic bacteria compared to the mastitis counterpart (Figure 2C).

### 3.3. Identification of the critical ruminal bacterial biomarkers

The beta diversity analysis and predicted phenotypes revealed the dissimilar ruminal bacteria existing between the two groups. Thus, the critical taxa associated with healthy and mastitis groups were then analyzed using the LEfSe algorithm with LDA > 2.5 as the bacterial biomarkers. The results identified 37 influential taxonomic clades, including 17 genera and 3 species (Figure 2D). The most impacted taxa in the healthy group were 12 genera (*Ruminococcaceae* UCG 014, *Eubacterium coprostanoligenes* group, *Eubacterium ruminantium* group, *Ruminococcus* 1, *Syntrophococcus*, *Dialister*, *Pseudobutyrivibrio*, *Desulfovibrio*, *Lachnoclostridium* 12, *Ruminococcaceae* UCG 007, *Peptostreptococcus*, *Mitsuokella*), and 2 species (*R. flavefaciens* and *B. longum* subsp. *longum*). Four genera (*Prevotella* 1, *Prevotellaceae* UCG001, *Prevotellaceae* UCG003, *Fibrobacter*) and one species (unidentified rumen bacterium RNF82) were the critical taxa in the mastitis group.

### 3.4. Correlation of mastitis parameters with the bacterial biomarkers

After identifying the bacterial biomarkers in both groups, we illustrated the correlation of mastitis parameters (SCC, NAGase, IL-6) with the bacterial biomarkers at the species level (Figure 2E). The species enriched in the healthy group, *R. flavefaciens* and *B. longum* subsp. *longum*, were negatively correlated with the levels of SCC and NAGase. Conversely, the species enriched in the mastitis group, the unidentified rumen bacterium RNF82, demonstrated positive correlations with the levels of SCC and NAGase. The relative abundance of the bacterial biomarkers related to the mastitis group was paralleled with the above findings. The mastitis group demonstrated a significantly lower relative abundance in the *R. flavefaciens* and *B. longum* subsp. *longum* (*p* < 0.05) (Figure 2F).

**Figure 3:**
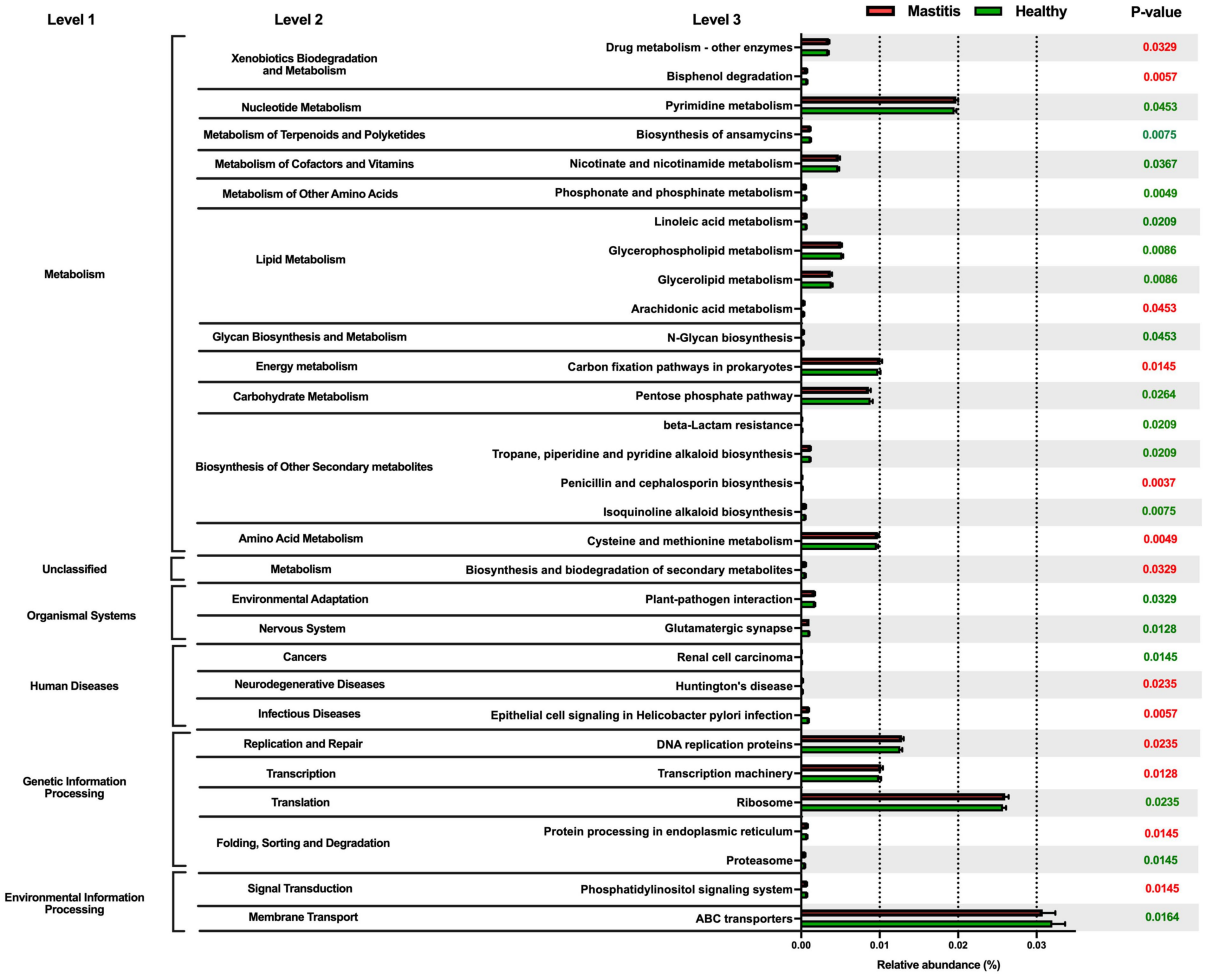
Comparison of the relative abundance of the PICRUSt functional prediction of the ruminal microbiota between healthy and mastitis groups. The results are presented as mean ± SEM (*n* = 15). Distinct gene categories were selected according to significant differences in the Kyoto Encyclopedia of Genes and Genomes (KEGG) pathway level 2 (Mann–Whitney U test, *p* < 0.01).

Phylogenetic investigation of communities by the reconstruction of unobserved states (PICRUSt) was applied to investigate the mastitis-associated functional profiles of microbiome communities (Figure 3). In the mastitis group, 13 functions were enriched, including xenobiotics biodegradation and metabolism, energy metabolism, amino acid metabolism by cysteine and methionine metabolism, neurodegenerative and infectious diseases, replication and repair, transcription and translation. The remaining 18 pathways were depleted, including nucleotide metabolism, lipid metabolism, glycan biosynthesis and metabolism, environmental adaptation, and vitamin B-related metabolic pathways.

### 3.5. Metabolomics analysis revealed a dissimilar ruminal metabolite composition

**Figure 4:**
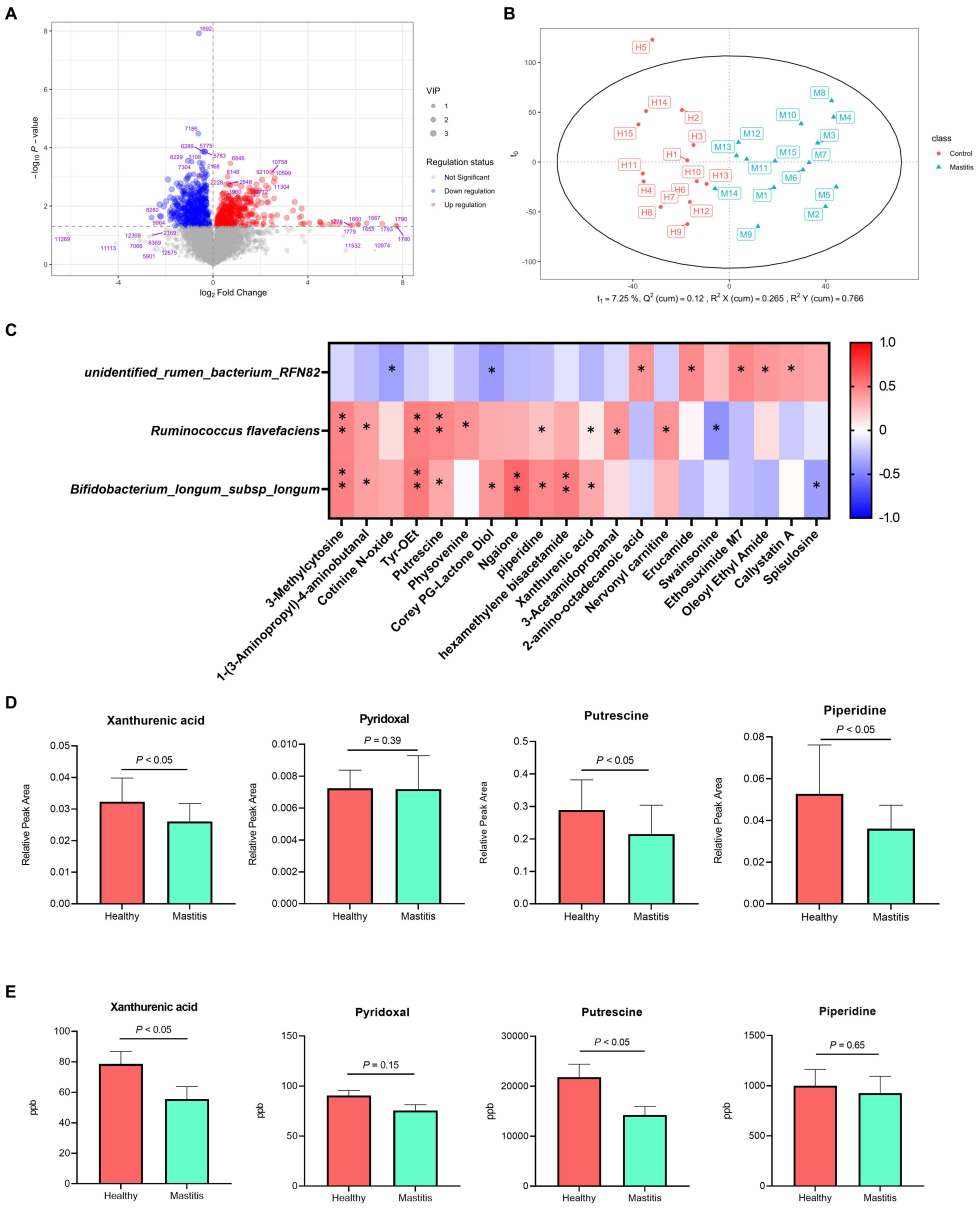
Different compositions of ruminal metabolites of healthy and mastitis cows. **(A)** Volcano plot of 1,181 compounds with log-transformed adjusted *p*-values and fold change. The green and red dots indicate significantly higher metabolites in the healthy and mastitis groups, respectively. **(B)** Orthogonal PLS-DA (oPLS-DA) plot based on the 1,181 compounds indicates significantly different metabolite compositions of the healthy and inflammatory groups. Ellipses represent 95% confidence intervals for each group. Every dot represents a single individual cow. **(C)** Reciprocal interrelationships between the ruminal microbiota and metabolome by Spearman’s correlation test shown at the species level. Orange and blue colors indicate positive and negative correlation coefficients, respectively. Symbols indicate the significant correlation between metabolites and biomarkers (*p < 0.05 and **p < 0.01). **(D)** The significant relative peak area and **(E)** concentration of metabolites positively correlated with *Ruminococcus flavefaciens* and *Bifidobacterium longum* subsp. *longum*.

In total, 1,181 compounds were identified through blasting, matching the mzCloud online database among 12,709 practicable peaks. A volcano plot showing the metabolite profile of the statistical significance (VIP > 1, FDR adjusted *p* < 0.05 threshold) against fold change revealed the ruminal metabolites with significant differences between the two groups (Figure 4A). Additionally, the oPLS-DA plot showed a clear separation between the healthy and mastitis groups, suggesting a dissimilar ruminal metabolite conformation (Figure 4B). Among identified metabolites, 65 metabolites were significantly different between the two groups (VIP > 1, FDR adjusted *p* < 0.05), of which, 29 were significantly lower in the mastitis group than those in the healthy group (Supplementary Table S1). Further analysis of the metabolic pathway using KEGG (the second level) identified 21 (methionine, putrescine, proline, piperidine, 5-hydroxyindoleacetic acid, N-(2-phenylethyl)-acetamide, 1-pyrroline, 3-acetamidopropanal, nervonyl carnitine, asparaginyl-alanine, triphenylsilanol, 4-aminophenylalanine, 2-phenylbutyramide, pyrophaeophorbide, linoleoyl ethanolamide, 6-pentadecyl salicylic acid, tyr-OEt, 1-(3-aminopropyl)-4-aminobutanal, hexamethylene bisacetamide, 1,5-diphenylcarbohydrazide, and ansamitocin P3) and 16 (carnitine, (alpha)-JWH 073 N-(3-hydroxybutyl) metabolite-d5, (6aR, 11aR)-3-hydroxy-8,9-dimethoxypterocarpan, 2-ethylpyrazine, erucamide, 2-amino-octadecanoic acid, Corey PG-lactone diol, juvenile hormone I, 2,3-dinor-8-iso-PGF2a, 2-acetylpyrazine, hydrocortamate, 9-hydroperoxy-10E, 12-octadecadienoic acid, ethyl 2-furanpropionate, oleoyl ethyl amide, osmundalactone, and 9-decynoic acid) metabolites involved in amino acid and lipid metabolism, respectively. Other pathways such as the metabolism of cofactors and vitamin, nucleotide metabolism, xenobiotic biodegradation metabolism, nicotinate metabolism, carbohydrate metabolism, energy metabolism, nervous system, and replication and repair were also identified.

### 3.6. Correlation of the ruminal microbiota and metabolome

The reciprocal inter-relationships between 20 ruminal microbial biomarkers and 65 metabolites, which were significantly different between the two groups, were analyzed with the Spearman’s correlation test. All the metabolites were significantly correlated with some of the critical bacterial biomarkers (*p* < 0.05) (Supplementary Figure S3). Among them, 3-methylcytosine, 1-(3-aminopropyl)-4-aminobutanal, putrescine, pyridoxal, xanthurenic acid, and Tyr-OEt, are related to amino acid metabolism, replication and repair, and metabolism of cofactors and vitamin (Supplementary Table S1) were significantly positively correlated with the bacterial species biomarkers in the healthy group, *R. flavefaciens* and *B. longum* subsp. *longum* (*p* < 0.05) (Figure 4C). The unidentified rumen bacterium RNF82, the bacterial biomarkers in the mastitis group, were negatively correlated with cotinine N-oxide, and Corey PG-lactone diol (*p* < 0.05) and positively correlated with 2-amino-octadecanoic acid, erucamide, and ethosuximide M7 (Figure 4C). By further quantifying the ruminal metabolites, the higher relative peak area of three metabolites, xanthurenic acid, pyridoxal, and putrescine in the healthy group (Figure 4D) demonstrated significantly higher concentrates in the ruminal fluid samples compared with the mastitis counterpart, verifying the ruminal metabolomic finding (Figure 4E).

### 3.7. Verification of the health-promoting effect of *Ruminococcus flavefaciens* and *Bifidobacterium longum* subsp. longum by FMT GF mice

**Figure 5:**
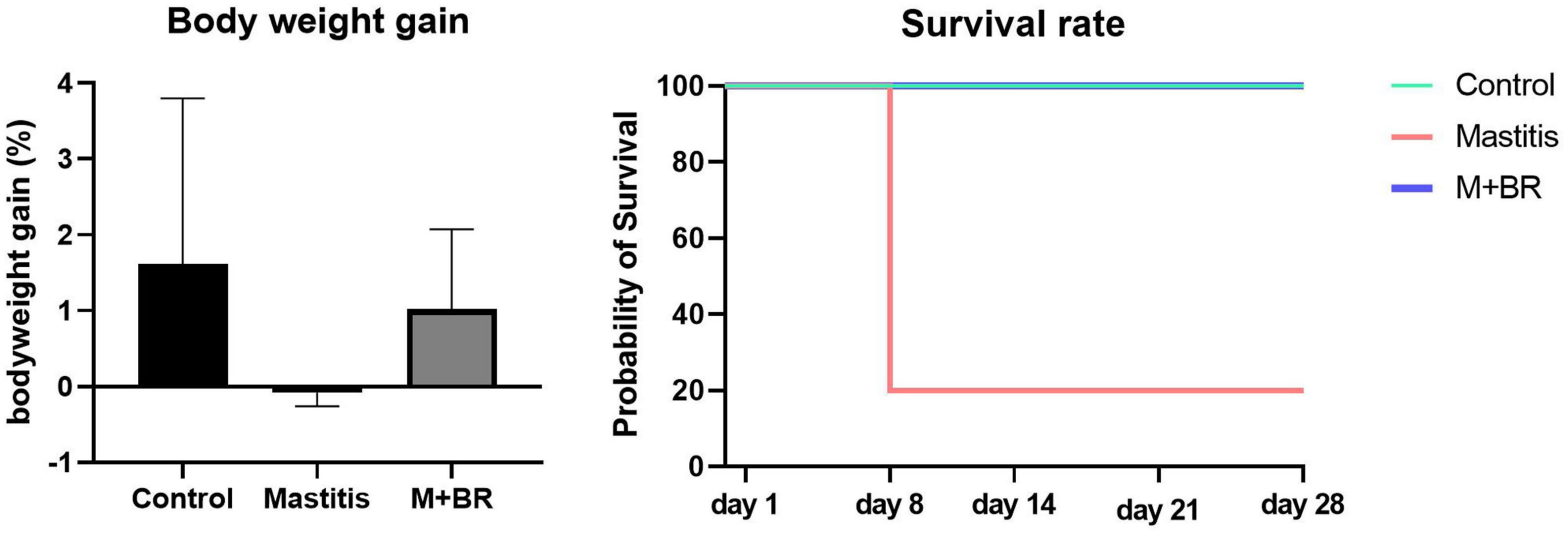
Survival rate and body weight gain after the fecal microbiota transplantation (FMT) test and bacterial biomarker supplements.

To verify the healthy promoting effect of *R. flavefaciens* and *B. longum* subsp. *longum*, fecal microbiota from the 15 mastitis and 15 healthy cows were, respectively, pooled and inoculated into GF mice. The results showed that the mice transplanting gut microbiota from mastitis cows could decrease the survival rate and bodyweight gain compared with that from healthy cows (Figure 5). Administration of *R. flavefaciens* and *B. longum* subsp. *longum* could increase body weight gain and reduce mortality rate.

### 3.8. Verification of inter-relationships between microbiota and metabolome *in vitro*

**Figure 6:**
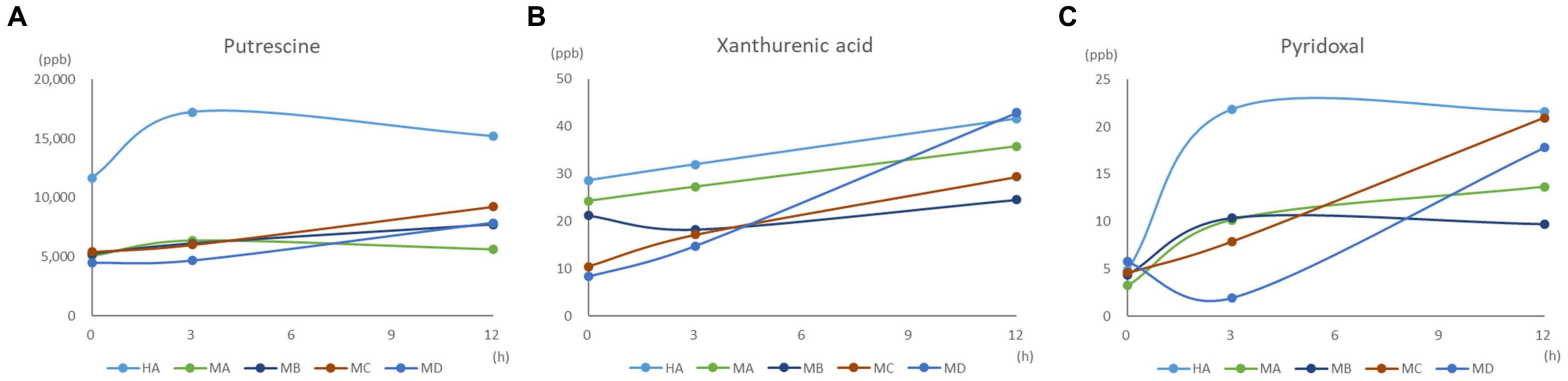
Effect of bacterial biomarker supplements on **(A)** putrescine, **(B)** xanthurenic acid, and **(C)** pyridoxal concentration during the 12 h *in vitro* fermentation.

We further investigated the inter-relationships between microbiota and metabolome by *in vitro* ruminal fermentation. First, the ruminal fluid pH value during a 12 h *in vitro* fermentation was above 6.0 in both the healthy and mastitis group. The mean pH was 6.72 (HA), 6.84 (HB), 6.83 (HC), 6.87 (HD), 6.95 (MA), 6.91 (MB), 7.00 (MC), and 7.02 (MD). The basic composition of the ruminal fluid in all groups had no significant change except NH_3_-N (Table 1). *R. flavefaciens* and *B. longum* subsp. *longum* addition demonstrated the trend to upregulate the levels of putrescine, xanthurenic acid, and pyridoxal in the mastitis ruminal fluid (Figure 6), which confirmed the inter-relationships between microbiota and metabolome. Additionally, the levels of total volatile fatty acids (VFA) were upregulated after inoculation with *R. flavefaciens* (HC and MC groups) and *R. flavefaciens* + *B. longum* subsp. *longum* (HD and MD groups) (Table 2). As supplement of *B. longum* subsp. *longum*, the relative amount of lactate would increase (HB and MB groups).

**Table 1:** Supplement of *Ruminococcus flavefacians* and *Bifidobacterium longum* subsp. *longum* on the digestibility of dietary nutrients in *in-vitro* fermentation.

Items^a^	HA^b^	HB	HC	HD	SE^c^	*p*-value
DM (%)	66.15	66.90	66.65	66.47	0.60	0.384
NDF (%)	59.76	60.45	60.58	60.02	0.52	0.153
ADF (%)	56.44	57.90	57.27	55.80	1.13	0.094
NH_3_-N (mg/dL)	17.50^c^	17.79^c^	30.97^b^	32.32^a^	2.86	0.001

	MA^d^	MB	MC	MD	SE	*p*-value
DM (%)	67.22	67.03	66.27	67.28	0.93	0.424
NDF (%)	59.81	59.23	58.25	58.58	1.94	0.683
ADF (%)	56.32	56.35	54.74	54.46	1.85	0.355
NH_3_-N (mg/dL)	16.39^d^	23.03^c^	29.03^b^	33.13^a^	4.15	0.001

a DM, dry matter; NDF, neutral detergent fibre; ADF, acid detergent fibre.

b HA, healthy group, without additional bacterial culture; HB, healthy group, with *B. longum* subsp. *longum*; HC, healthy group, with *R. flavefaciens*; HD, healthy group, with *B. longum* subsp. *longum* and *R. flavefaciens*.

c SE, standard error.

d MA, mastitis group, without additional bacterial culture; MB, mastitis group, with *B. longum* subsp. *longum*; MC, mastitis group, with *R. flavefaciens*; MD, mastitis group, with *B. longum* subsp. *longum* and *R. flavefaciens*.

^a–d^Values within a row with different superscripts differ significantly at *p* < 0.05.

**Table 2:** Effects of bacterial biomarker supplements on the concentration of volatile fatty acids (VFA) and lactic acid in healthy and mastitis groups after 12 h of *in vitro* fermentation.

Items^a^	HA^b^		HB		HC		HD		SE^c^	*p*-value
VFA (mM)										
Acetate	23.26^b^	24.57^b^	30.28^a^	31.89^a^	1.27	0.001
Propionate	7.86	7.61	8.31	8.95	0.74	0.109
Butyrate	4.16	4.23	4.53	4.97	0.48	0.120
Isobutyrate	0.10^b^	0.13^b^	0.14^b^	0.18^a^	0.03	0.010
Valerate	0.29	0.44	0.46	0.48	0.13	0.191
A/P	2.96^c^	3.23^b^	3.66^a^	3.61^a^	0.31	0.026
Total VFA	39.79^d^	42.73^c^	47.09^b^	50.64^a^	1.85	<0.0001
Lactate	3.70^b^	5.34^a^	2.86^c^	3.67^b^	0.60	0.001

	MA^d^		MB		MC		MD		SE	*p*-value
VFA (mM)										
Acetate	22.32	28.57	29.08	28.63	3.64	0.065
Propionate	7.85^b^	7.93^b^	10.81^a^	8.38^b^	1.27	0.019
Butyrate	4.67	4.20	5.26	5.93	0.88	0.079
Isobutyrate	0.08^b^	0.14^ab^	0.15^ab^	0.17^a^	0.02	0.001
Valerate	0.30	0.34	0.32	0.33	0.05	0.693
A/P	2.94	3.72	2.68	3.46	0.93	0.1797
Total VFA	37.69^c^	44.93^b^	48.13^a^	46.84^ab^	4.14	0.017
Lactate	2.12^c^	3.35^a^	2.11^c^	2.93^b^	0.42	0.003

a VFA, volatile fatty acid; A/P, acetate/propionate.

b HA, healthy group, without additional bacterial culture; HB, healthy group, with *B. longum* subsp. *longum*; HC, healthy group, with *R. flavefaciens*; HD, healthy group, with *B. longum* subsp. longum and *R. flavefaciens*.

c SE, standard error.

d MA, mastitis group, without additional bacterial culture; MB, mastitis group, with *B. longum* subsp. *longum*; MC, mastitis group, with *R. flavefaciens*; MD, mastitis group, with *B. longum* subsp. *longum* and *R. flavefaciens*.

^a–d^Values within a row with different superscripts differ significantly at *p* < 0.05.

## 4. Discussion

In the present study, we verified the inter-relationship of microbiome and metabolome biomarkers to potentially promote the health of dairy cows. First, a stable and resilient core microbiota in the ruminal fluid of the lactating cow w/wo mastitis was observed based on the results of the Venn diagram, Shannon, Chao1, and the relative abundance of the taxa at the different levels. The genera *Prevotella* 1, *Ruminococcaceae* NK4A214 group, and *Christensenellaceae* R7 group, the top three predominant genera in both healthy and mastitis groups, were also the abundant genera in the rumens of lactating cows, dry period cows (32), yak (33), and mastitis cows (7). These three genera play important roles in protein degradation and lipid biohydrogenation (3), SCFA production by the breakdown of fibrous plants (34), and microbial inhibiting activities (35). The *Ruminococcaceae* NK4A214 group was also positively correlated to milk total solids in lactating cows (36).

Although the bovine rumen demonstrated a highly similar microbial composition, the differences in taxa and individual bacterial abundance still existed between the lactating cows w/wo mastitis, which could effectively distinguish these two groups using the PLS plot and unweighted UniFrac. The ruminal microbiota of mastitis cows was characterized by high Gram-negative and potentially pathogenic bacteria. KEGG pathways with higher abundance in mastitis cows, including xenobiotics acid biodegradation and metabolism, energy metabolism, replication and repair, transcription, translation, and infectious disease, can be related to the inflammation and mucosa repairing in cows (37). The genera *Prevotella* (*Prevotella* 1, *Prevotellaceae* UCG001, *Prevotellaceae* UCG003) and *Fibrobacter* were the biomarkers associated with mastitis cows. *Prevotella* with diverse isoforms is crucial for ruminal fermentation (38); however, the high abundance of this genus was associated with high-grain feed (39) as well as acidosis (40). The studies in humans and animals have connected the increased abundance of *Prevotella* species at mucosal sites with localized and systemic inflammation disease due to enhancing T helper type 17 (Th17)-mediated mucosal inflammation via IL-8, IL-6, and CCL20 stimulation (41). The increasing serum IL-6 in mastitis cows in this study may partially support the ruminal dysbiosis leading to systemic inflammatory effect.

Conversely, the ruminal microbiota associated with healthy cows was categorized by high-stress tolerant, Gram-positive, and SCFA producing bacteria. This finding was later confirmed by *in vitro* ruminal fermentation. Increasing VFA and SCFA with healthy ruminal liquid were due to the high SCFA producing bacteria. The reduction of SCFA producing bacteria in ruminal fluid (7) and feces (9) of mastitis cows has been reported. Higher KEGG pathway in lipid and carbohydrate metabolisms in healthy cows also suggested reduced lipid and carbohydrate metabolic activities of the gut microbiota in mastitis cows. Our finding was in line with a study indicating that carbon metabolism was less abundant in mastitis cows (9). Downregulation of carbohydrate metabolism may alter the glucose and carbohydrate balance in the body (42), which affects the energy for maintenance, growth, and production in farm animals (43).

The biomarkers identified in the healthy group, including the genera *Ruminococcus*, *Eubacterium*, *Lachnoclostridium*, and *Pseudobutyrivibrio*, known as cellulose and fiber degraders (44), associated with high-yield cows (45). The genus *Syntrophococcus* has been reported to utilize sugars and H_2_-CO_2_-using methanogens as electron donors to produce acetate and as an electron acceptor, respectively (46). At the species level, *R. flavefaciens* could modify the abundance of other cellulolytic bacterial populations (47) and improve the feed efficacy for ruminants (48). Another species, *B. longum* subsp. *longum*, a biomarker in the healthy group, was also reported to stabilize gut microbiota and improve the intestinal environment (49). Both species recognized in the healthy cow could be potential probiotics to promote animal health, which warrants further investigation.

From the *in vitro* ruminal fermentation, additional inoculation of *R. flavefaciens* and *B. longum* subsp. *longum* could significantly impact the levels of NH_3_-N, total VFA, and VFA profiles in both healthy and mastitis groups. NH_3_-N is the main nitrogen source used by microbes to synthesize amino acid and peptide bonds for growth (50). *Ruminobacter* spp. is a hyper-ammonia producing (HAP) bacteria (51). The increase in NH_3_-N could be explained by the inoculation of *R. flavefaciens*, which leads to an increased population of HAP bacteria and deaminase activity. Total VFA and VFA profiles are important products of the bacterial fermentation activity in the rumen, which have emerged as key regulators in intestinal and energy homeostasis regulation (52). *R. flavefaciens* participates in the butyrate metabolic pathway (53, 54). Upregulating the VFA concentration has been reported in repeated ruminal dosing of *R. flavefaciens* in dairy cows (47). The increase in VFA suggested an upregulating deamination activity (55) by the addition of *R. flavefaciens*. The increase in lactate is expected with additional *B. longum* subsp. *longum*, a lactic acid producer. The administration of lactic acid bacterial probiotics is thought to help rumen microbiota adapt to the presence of lactic acid (56) and prevent lactate accumulation in the rumen (57). Nevertheless, the increase in VFA did not have much physiological impact as the rumen pH due to shifting the microbiota to lactate-consuming bacteria.

FMT verified the inter-relationship among gut dysbiosis, systemic inflammatory effect, and health-promoting ability of two microbial biomarkers, *R. flavefaciens* and *B. longum* subsp. *longum*. Ma et al. (9) found that FMT from diseased cows caused mastitis-like symptoms in mice by shifting the murine intestinal microbiota. Although the acute inflammation led to mice mortality after FMT with mastitis feces, which could not provide solid evidence between mastitis and the dysbiosis of ruminal microbiota, the findings confirmed the impact of gut microbiota as one potential parameter affecting dairy cow health. The increase in the survival rate after FMT of mastitis feces with *R. flavefaciens* and *B. longum* subsp. *longum* also supported the potential efficacy of microbial biomarkers as probiotic treatment, which may point to a new health-promoting and disease-preventing approach.

Besides the microbiota, bacterial products, in turn of metabolome, were also a key factor involved in bovine health and systemic disease outcomes. Three metabolites (i.e., putrescine, xanthurenic acid, and pyridoxal) were verified by further *in vitro* ruminal fermentation and HPLC qualitative analysis, which were positively correlated with two species biomarkers, *R. flavefaciens* and *B. longum* subsp. *longum*. Putrescine, a biogenic amine produced from the decarboxylation of amino acids by decarboxylase in certain intestinal microorganisms (58), has been found in rumen fluid of healthy animals, which was in line with our findings. Putrescine has antioxidant and anti-inflammatory attributes (59) and is involved in the growth of tissues and organs (60). Xanthurenic acid, a non-indolic catabolite of tryptophan and a metabolite of the kynurenine pathway (61), demonstrated profound effects on the gut microbial composition, host-microbiome interface, and host immune system–intestinal microbiota interactions. Pyridoxal is one of the natural forms available of vitamin B6, supplied either in the diet or by rumen or intestinal symbiosis for bovine species. Vitamin B6 participates in DNA, RNA and protein synthesis. Mastitis is associated with a vitamin B metabolism disorder in intestinal microbiota (9). The upregulated putrescine, xanthurenic acid, and pyridoxal in ruminal fluid after adding *R. flavefaciens* and *B. longum* subsp. *longum* suggested a health effect on modulating intestinal homeostasis and damage repair.

## 5. Conclusion

Although the bovine rumen possesses a strong core microbial composition, we proved that minor microbiota shifting caused by mastitis could affect the health of dairy cows. This influence is not only because of the rumen microbiota but the downstream microbiome produced by microbiota also plays an important role in health. To the best of our knowledge, this study is the first to verify the inter-relationship of microbiome and metabolome biomarkers for the potential to promote health in dairy cows. Two species, *R. flavefaciens* and *B. longum* subsp. *longum*, with three metabolites, putrescine, xanthurenic acid, and pyridoxal, were identified in the ruminal fluid, which may point to a new direction to promote health and prevent disease in dairy cattle.

## Data availability statement

The original contributions presented in the study are included in the article/Supplementary material, further inquiries can be directed to the corresponding author.

## Ethics statement

The animal studies were approved by Institutional Animal Care and Use Committee of National Taiwan University (IACUC approval no: NTU-107-EL-00221). The studies were conducted in accordance with the local legislation and institutional requirements. Written informed consent was obtained from the owners for the participation of their animals in this study.

## Author contributions

S-TC and M-JC conceived and designed the experiments. J-CH, S-TC, K-YL, Y-TH, and S-TH performed the experiments. J-CH, K-YL, Y-TH, and S-HC analyzed the data. J-CH, S-TC, and M-JC wrote and revised the manuscript. All authors contributed to the article and approved the submitted version.

## Funding

This research was funded by the Ministry of Science and Technology of Taiwan (MOST 105-2313-B-002-041-MY3 and MOST 109-2321-B-002-054).

## Conflict of interest

S-HC was employed by Biotools Co. Ltd.

The remaining authors declare that the research was conducted in the absence of any commercial or financial relationships that could be construed as a potential conflict of interest.

## Publisher’s note

All claims expressed in this article are solely those of the authors and do not necessarily represent those of their affiliated organizations, or those of the publisher, the editors and the reviewers. Any product that may be evaluated in this article, or claim that may be made by its manufacturer, is not guaranteed or endorsed by the publisher.

## Footnotes

^1^ https://www.metaboanalyst.ca
