# Supplementary figures and images for: In vitro ruminal fermentation and cow-to-mouse fecal transplantations verify the inter-relationship of microbiome and metabolome biomarkers: potential to promote health in dairy cows
Source: Front Vet Sci. 2023 Aug 17;10:1228086. doi: 10.3389/fvets.2023.1228086 (PMC10469932; doi:10.3389/fvets.2023.1228086)

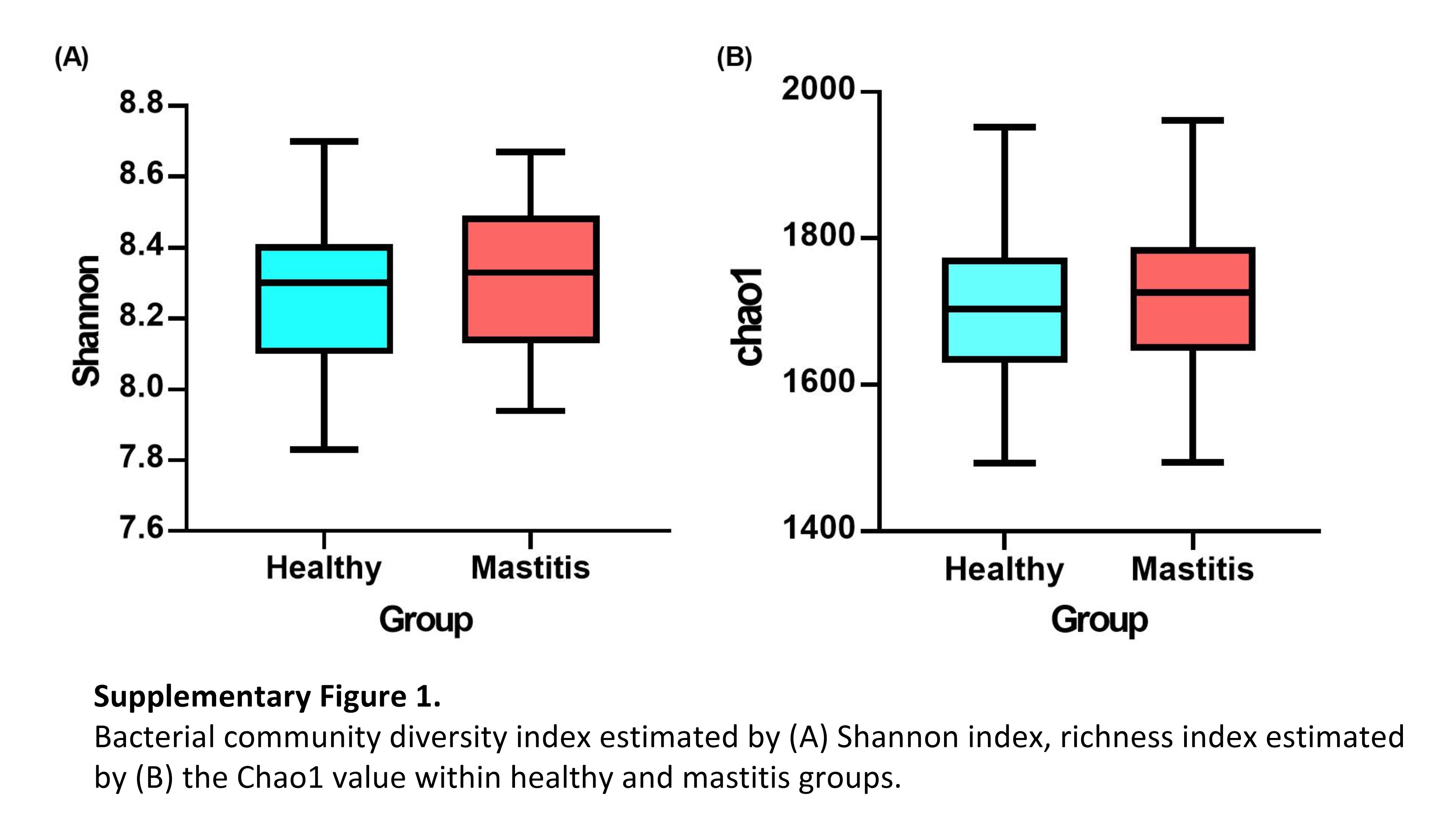

Supplement: Supplementary file 1 [file Image_1.jpg]

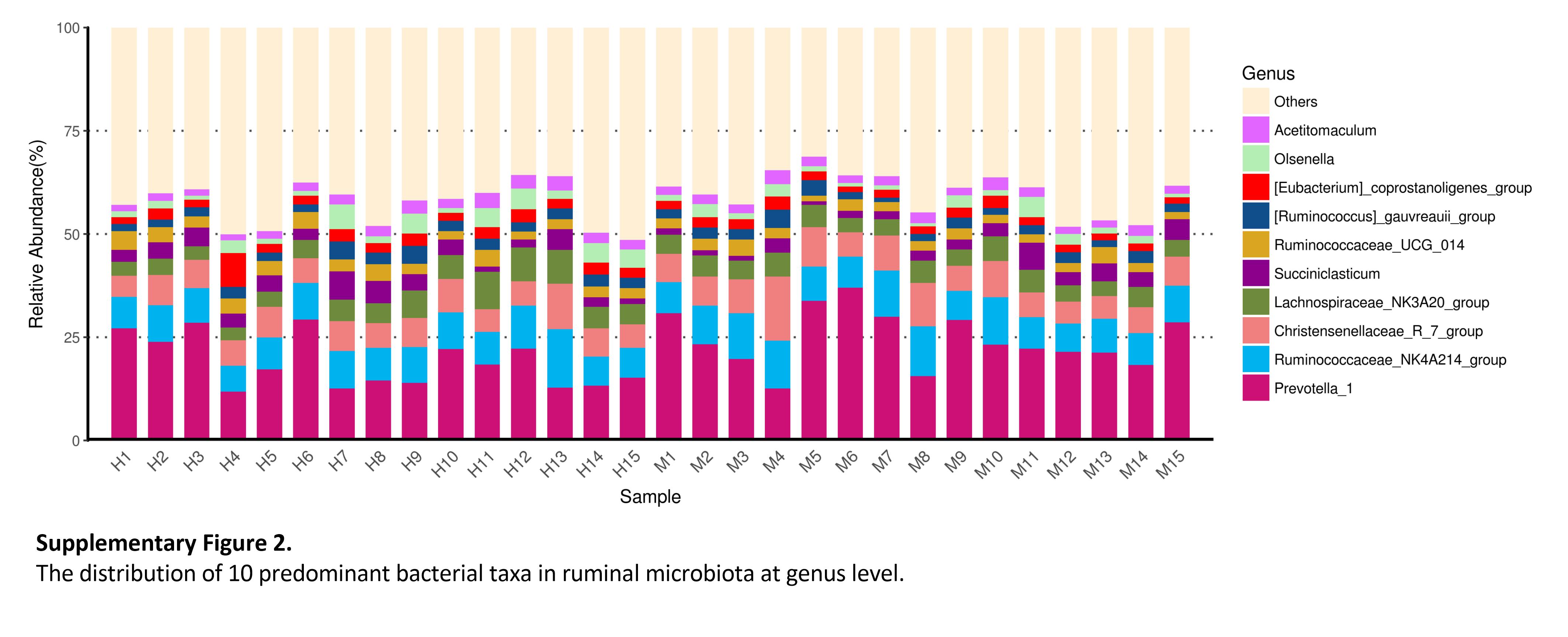

Supplement: Supplementary file 2 [file Image_2.jpg]

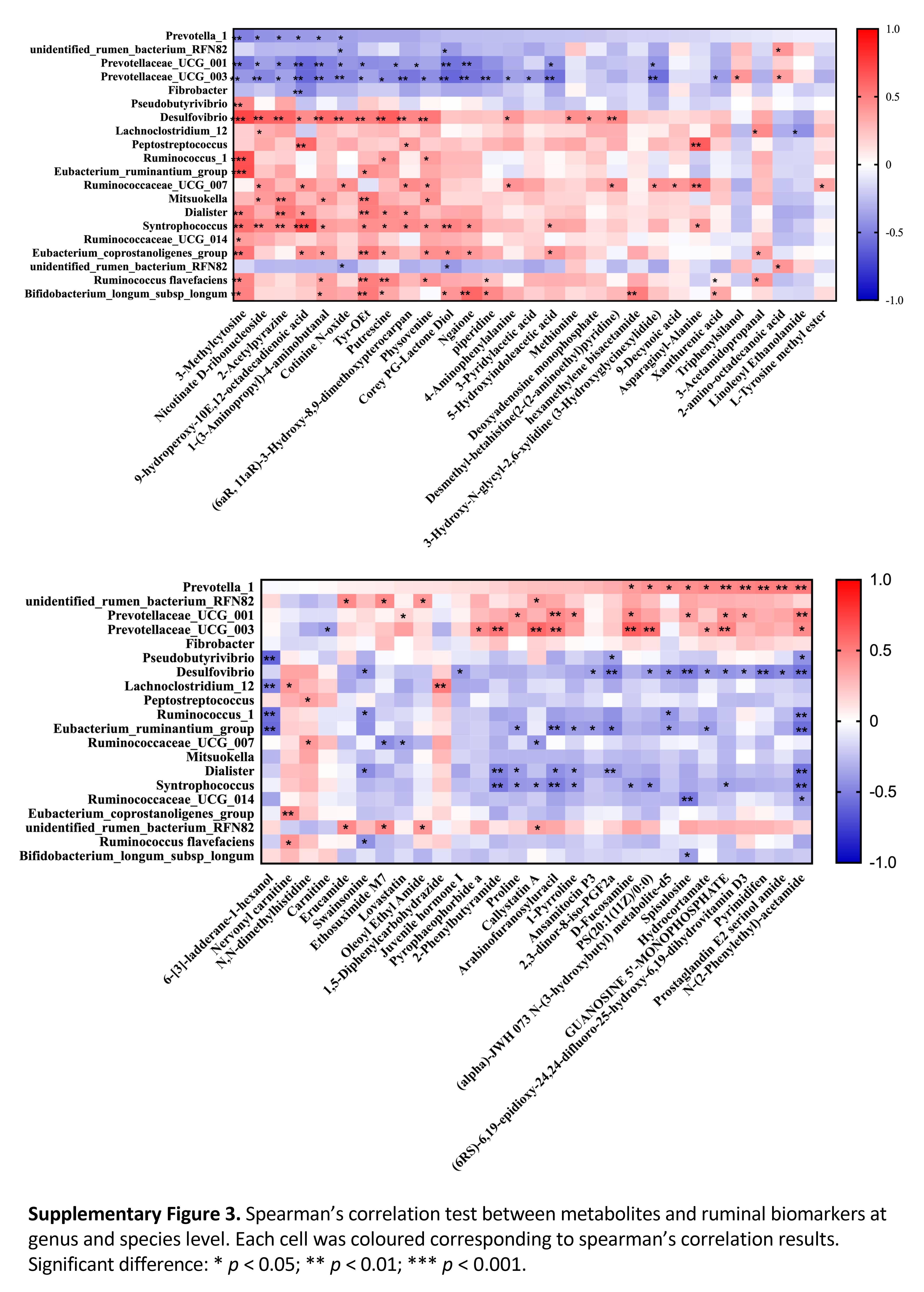

Supplement: Supplementary file 3 [file Image_3.jpg]
