# Supplementary material for: In vitro ruminal fermentation and cow-to-mouse fecal transplantations verify the inter-relationship of microbiome and metabolome biomarkers: potential to promote health in dairy cows
Source: Front Vet Sci. 2023 Aug 17;10:1228086. doi: 10.3389/fvets.2023.1228086 (PMC10469932; doi:10.3389/fvets.2023.1228086)
Supplement: Supplementary file 4 [file Table_1.docx]

**Supplementary Table 1.**

65 metabolites with significant difference between groups in ruminal fluid samples. The Student’s t-test was used to compare the signal intensities of metabolites profiles between the healthy and mastitis groups (*p* < 0.05, VIP > 1). Significant difference: * *p* < 0.05; ** *p* < 0.01; *** *p* < 0.001.

| ID | Metabolite | Healthy  (n = 15) | Mastitis  (n = 15) | VIP | Significance | Pathway |
| --- | --- | --- | --- | --- | --- | --- |
|  |  | Mean | Mean |  |  |  |
| 2 | Nicotinate D-ribonucleoside | 3.926 | 1.868 | 2.631 | ** | Metabolism of cofactors and vitamin |
| 20 | Methionine | 1.361 | 1.167 | 1.702 | * | Amino acid metabolism |
| 26 | Putrescine | 28.904 | 21.510 | 2.405 | * | Amino acid metabolism |
| 27 | Guanisine 5'-monoposphate | 1.970 | 5.406 | 2.061 | ** | Nucleotide metabolism |
| 29 | Proline | 77.935 | 141.399 | 2.050 | * | Amino acid metabolism |
| 39 | Carnitine | 1.436 | 1.145 | 1.641 | * | Lipid metabolism |
| 43 | Deoxyadenosine monophosphate | 1.466 | 0.710 | 1.726 | * | Nucleotide metabolism |
| 67 | Piperidine | 5.275 | 3.603 | 1.698 | * | Amino acid metabolism |
| 74 | Xanthurenic acid | 3.229 | 2.609 | 1.706 | * | Metabolism of cofactors and vitamin |
| 82 | L-Tyrosine methyl ester | 4.277 | 3.210 | 1.560 | * | Transcription |
| 92 | 5-Hydroxyindoleacetic acid | 1.870 | 1.447 | 1.655 | ** | Amino acid metabolism |
| 100 | N-(2-Phenylethyl)-acetamide | 1.020 | 1.798 | 2.725 | ** | Amino acid metabolism |
| 104 | Lovastatin | 0.104 | 0.282 | 1.789 | ** | Xenobiotics biodegradation metabolism |
| 147 | 1-Pyrroline | 1.056 | 1.881 | 2.165 | * | Amino acid metabolism |
| 154 | 3-Acetamidopropanal | 3.209 | 2.482 | 1.072 | * | Amino acid metabolism |
| 165 | NICOTINYL | 0.247 | 0.577 | 1.157 | * | Nicotinate metabolism |
| 171 | 5-Hydroxy-3,4-dihydrocarbostyryl | 0.471 | 0.388 | 1.687 | ** | Carbohydrates metabolism |
| 174 | Nervonyl carnitine | 2.828 | 2.422 | 1.392 | * | Amino acid metabolism |
| 177 | (alpha)-JWH 073 N-(3-hydroxybutyl) metabolite-d5 | 1.883 | 2.936 | 2.366 | * | Lipid metabolism |
| 192 | Cotinine N-oxide | 1.949 | 1.142 | 1.718 | * | Nicotinate metabolism |
| 221 | (6aR, 11aR)-3-Hydroxy-8,9-dimethoxypterocarpan | 3.926 | 1.868 | 2.631 | *** | Lipid metabolism |
| 222 | Pyrimidifen | 0.313 | 0.810 | 2.459 | * | Energy metabolism |
| 237 | 3-Hydroxy-N-glycyl-2,6-xylidine (3-Hydroxyglycinexylidide) | 10.584 | 8.448 | 1.478 | * | Function unknown |
| 241 | D-Fucosamine | 0.476 | 0.967 | 2.302 | * | Carbohydrates metabolism |
| 245 | 2-Ethylpyrazine | 0.614 | 0.560 | 1.458 | * | Lipid metabolism |
| 268 | Spisulosine | 0.309 | 0.405 | 2.339 | * | Xenobiotics biodegradation metabolism |
| 269 | Physovenine | 9.989 | 3.984 | 2.054 | * | Metabolism of terpenoids |
| 274 | 6- [3]-ladderane-1-hexanol | 0.548 | 0.698 | 1.505 | * | Carbohydrates metabolism |
| 285 | Asparaginyl-Alanine | 2.025 | 0.943 | 1.724 | * | Amino acid metabolism |
| 298 | Prostaglandin E2 serinol amide | 0.330 | 0.544 | 2.176 | * | Nervous system |
| 314 | Erucamide | 43.545 | 63.777 | 1.120 | * | Lipid metabolism |
| 316 | Triphenylsilanol | 1.105 | 1.571 | 1.558 | * | Amino acid metabolism |
| 317 | 2-amino-octadecanoic acid | 0.283 | 0.780 | 2.697 | * | Lipid metabolism |
| 338 | 4-Aminophenylalanine | 3.019 | 2.096 | 1.475 | * | Amino acid metabolism |
| 348 | 2-Phenylbutyramide | 0.409 | 0.648 | 1.621 | * | Amino acid metabolism |
| 349 | 3-Pyridylacetic acid | 0.513 | 0.407 | 1.547 | * | Nicotinate metabolism |
| 360 | Arabinofuranosyluracil | 2.193 | 3.180 | 2.020 | ** | Carbohydrates metabolism |
| 364 | 3-Methylcytosine | 2.634 | 1.533 | 2.232 | * | Replication and repair |
| 395 | Desmethyl-betahistine(2-aminoethyl pyridine) | 1.334 | 0.940 | 1.898 | * | Nucleotide metabolism |
| 404 | Corey PG-Lactone Diol | 2.955 | 1.922 | 1.505 | ** | Lipid metabolism |
| 408 | Juvenile hormone I | 1.541 | 3.307 | 2.754 | * | Lipid metabolism |
| 430 | 2,3-dinor-8-iso-PGF2a | 1.620 | 1.952 | 2.686 | * | Lipid metabolism |
| 431 | Swainsonine | 1.688 | 2.282 | 2.291 | * | Amino acid compound |
| 437 | Pyrophaeophorbide | 17.477 | 23.595 | 1.827 | ** | Amino acid metabolism |
| 447 | Ethosuximide M7 | 1.745 | 2.588 | 1.394 | ** | Nervous system |
| 494 | 2-Acetylpyrazine | 1.498 | 1.050 | 2.807 | ** | Lipid metabolism |
| 500 | Hydrocortamate | 0.227 | 0.264 | 2.145 | * | Lipid metabolism |
| 507 | Linoleoyl Ethanolamide | 0.370 | 0.823 | 2.487 | * | Amino acid metabolism |
| 510 | 6-pentadecyl Salicylic Acid | 0.198 | 0.417 | 1.650 | * | Amino acid metabolism |
| 519 | Tyr-OEt | 0.246 | 0.110 | 1.047 | * | Amino acid metabolism |
| 568 | 9-hydroperoxy-10E,12-octadecadienoic acid | 16.234 | 10.985 | 1.108 | * | Lipid metabolism |
| 574 | Ethyl 2-furanpropionate | 1.245 | 0.906 | 1.485 | * | Lipid metabolism |
| 577 | 1-(3-Aminopropyl)-4-aminobutanal | 61.088 | 30.872 | 2.043 | * | Amino acid metabolism |
| 598 | Ngaione | 0.502 | 0.365 | 1.382 | * | Nicotinate metabolism |
| 609 | PS (20:1(11Z)/0:0) | 0.208 | 0.301 | 2.233 | ** | Membrane transport |
| 624 | hexamethylene bisacetamide | 1.578 | 1.147 | 1.027 | * | Amino acid metabolism |
| 670 | Oleoyl Ethyl Amide | 1.665 | 2.367 | 1.252 | * | Lipid metabolism |
| 711 | Quinagolide sulfate | 0.553 | 0.349 | 1.802 | * | Xenobiotics biodegradation metabolism |
| 730 | 1,5-Diphenylcarbohydrazide | 2.046 | 1.682 | 1.597 | ** | Amino acid metabolism |
| 745 | Callystatin A | 0.146 | 0.256 | 1.968 | * | Signal transduction |
| 776 | N, N-dimethylhistidine | 1.289 | 0.892 | 1.316 | * | Biosynthesis metabolism |
| 777 | (6RS)-6,19-epidioxy-24,24-difluoro-25-hydroxy-6,19-dihydrovitamin D3 | 0.241 | 0.300 | 2.241 | * | Metabolism of cofactors and vitamin |
| 822 | Osmundalactone | 1.510 | 1.017 | 1.963 | * | Lipid metabolism |
| 824 | 9-Decynoic acid | 1.547 | 1.230 | 1.375 | * | Lipid metabolism |
| 851 | Ansamitocin P3 | 2.394 | 3.126 | 1.978 | * | Amino acid metabolism |
